# 3D Visual Data-Driven Spatiotemporal Deformations for Non-Rigid Object Grasping Using Robot Hands

**DOI:** 10.3390/s16050640

**Published:** 2016-05-05

**Authors:** Carlos M. Mateo, Pablo Gil, Fernando Torres

**Affiliations:** 1 Computer Science Research Institute, University of Alicante, San Vicente del Raspeig, Alicante 03690, Spain; carlos.mateo@ua.es; 2 Physics, Systems Engineering and Signal Theory Department, University of Alicante, San Vicente del Raspeig, Alicante 03690, Spain; fernando.torres@ua.es

**Keywords:** visual perception, vision algorithms for grasping, 3D-object recognition, sensing for robot manipulation

## Abstract

Sensing techniques are important for solving problems of uncertainty inherent to intelligent grasping tasks. The main goal here is to present a visual sensing system based on range imaging technology for robot manipulation of non-rigid objects. Our proposal provides a suitable visual perception system of complex grasping tasks to support a robot controller when other sensor systems, such as tactile and force, are not able to obtain useful data relevant to the grasping manipulation task. In particular, a new visual approach based on RGBD data was implemented to help a robot controller carry out intelligent manipulation tasks with flexible objects. The proposed method supervises the interaction between the grasped object and the robot hand in order to avoid poor contact between the fingertips and an object when there is neither force nor pressure data. This new approach is also used to measure changes to the shape of an object’s surfaces and so allows us to find deformations caused by inappropriate pressure being applied by the hand’s fingers. Test was carried out for grasping tasks involving several flexible household objects with a multi-fingered robot hand working in real time. Our approach generates pulses from the deformation detection method and sends an event message to the robot controller when surface deformation is detected. In comparison with other methods, the obtained results reveal that our visual pipeline does not use deformations models of objects and materials, as well as the approach works well both planar and 3D household objects in real time. In addition, our method does not depend on the pose of the robot hand because the location of the reference system is computed from a recognition process of a pattern located place at the robot forearm. The presented experiments demonstrate that the proposed method accomplishes a good monitoring of grasping task with several objects and different grasping configurations in indoor environments.

## 1. Introduction

Robot grasping and intelligent manipulation in unstructured environments require the planning of movement according to objects’ properties and robot kinematics, as is discussed in [1] and a suitable perception of environment using sensing systems such as visual, tactile, force or combinations of them. In addition, working with the knowledge of the model’s uncertainties can be useful when the objects and/or their properties are unknown [2]. In the past, most of the work in robot grasping was focused on providing movements and points to grasp. However, current methods are concerned with adapting the gripper or robot hand to objects and the environment [3]. Bohg *et al.* [4] give an overview of the methodologies and existing problems relating to object-grasp representation (local or global), prior-object knowledge (known and unknown) and its features (2D, 3D or multimodal information) and the type of hand used (gripper and multi-fingered). In other works, tactile sensors were used to provide information about an object’s properties through physical contact. Thus, Chitta *et al.* [5] presented a tactile approach to classify soft and hard objects such as bottles, with or without liquids, according to texture measurements, as well as the hardness and flexibility properties of objects. Furthermore, Yousef *et al.* [6] provided a review of tactile sensing solutions based on resistive techniques, the predominant choice for grasping objects.

Although the detection of problems such as slippage and grip force can be controlled using hand kinematics and tactile sensors, the coordinated control among robot-hand joints and tactile sensors is not good enough to perform grasping complex tasks for deformable objects [7]. It is recommended to integrate other sensors that imitate human dexterity, that is, systems based on real-time visual inspection of the task. Therefore, this research involved the implementation of a new visual sensing system based on range imaging technology so as to increase the available sensing data and facilitate the completion of complex manipulation tasks using visual feedback. This work is motivated by the necessity of using other different sensors that tactile sensors for controlling the grasping process of elastic objects. In fact, empirical experiments prove as tactile sensors often supply pressure values close to zero in the contact points of elastic object surface when the object is being deformaded during a task grasping. In this case, the pressure is not adequate to control the grasping process. In general, the kinematics of robot systems and tactile feedback obtained directly from tactile sensors are both used to perform intelligent, dexterous manipulation but it is rare to find practical work in which visual sensors are used to check grasping tasks using robots. Nevertheless, a suitable sensing system, such as in [8], that combines tactile, force and visual sensors, allow us to adapt the grasping task in order to detect errors that can cause grasping failure . Furthermore, it evaluates the effectiveness of the manipulation process by improving the grasp of deformable objects. Similarly, Li *et al.* [9] proposed a controller based on visual and tactile feedback to perform robust manipulation, even in the presence of finger slippage, although flexible objects are not considered, only rigid ones. In our method, the physical model of the skeleton of multi-finger hand for the grasping process is estimated from the kinematics and spatial location of the forearm. Our method does not depend on the pose of the multi-finger hand because the location of the reference system is computed from a recognition process of a pattern located place at the robot forearm. The presented experiments demonstrate that the proposed method accomplishes a good monitoring of grasping tasks with several objects and different grasping configurations in indoor environments. Additionally, the features of the objects are changing in shape, size, surface reflectance and elasticity of the material of which they were made. Our research focuses on checking robot-grasping tasks using 3D visual data from a RGBD camera. Therefore, the approach presented here provides a new visual sensing system in which the grasped non-rigid object surface is supervised to prevent problems like slippage and lack of contact among the fingers, caused by irregular deformations of the object. The main goal of our work is to implement a visual perception system to achieve a robust robotic manipulation system by means of object surface analysis throughout the grasping process. Specific aspects relating to how the robot controller uses the data generated by our visual system and how those are combined with tactile or another information will be addressed in other works. The proposed method of 3D visual inspection for the manipulation of flexible objects has been tested in real experiments where flexible household objects are manipulated by a robot hand. These objects are made of different materials and have different size, shape and texture. Our method uses colour and geometrical information to detect an object in an environment and track its grasping, even if the object has unknown elasticity and flexibility properties. In comparison with other approaches, our method does not use deformations models of both objects and materials and, it also works well with both planar and 3d household objects in real time.

The paper is organized as follows: Section 2 is focused on the analysis of several related works with visual perception for attending to robotic grasping processes. Section 3 describes the proposed visual system for the surveillance of grasping tasks; details on the equipment and facilities used are presented here. Section 4 presents the specification, design and implementation of the recognition method for detection of an object and its surface, and also presents the theoretical principles and fundamentals for modelling grasping tasks, using the kinematics of the robot hand and the detected object which is being manipulated. Section 5 and Section 6 present the novel method for measuring the deformation caused by pressure that occur during the grasping of flexible objects. Finally, Section 7 and Section 8 describe the tests, outlines our approach and discuss the results of real grasping tasks using three objects with different physical properties, such as size, shape, material, texture and colour as well as reflectance properties due to the material of manufacturing. Additionally, an statistical analysis of experiments is realised to show the behaviour of our method.

## 2. Related Works

In the past, visual systems were used quite successfully for the manipulation of rigid objects, both for recognition [10] and for the location of an object [11]. This developments and applications of intelligent robot manipulation involve methods and approaches aimed at achieving an object classification and recognition goal. Nevertheless, 3D visual systems are combined with others to measure objects’ shapes and the elasticity of deformable objects. We consider elasticity to be the ability of an object to recover its normal shape after being compressed. In [12] is shown an example of an embedded multisensor based on a CCD camera and a tactile membrane, which was designed to determine the contact area and forces’ distribution in order to classify materials and their deformation properties.

Furthermore, other works are focused on detecting and tracking the deformation from the fusion of 2D images with force data [13,14]. Later, Khalil *et al.* [15] used stereoscopic vision to build a 3D surface mesh from contours and colour in order to discover the deformation of non-rigid objects, and Leeper *et al.* [16] used a low-cost stereo sensor mounted on the gripper to estimate grasp poses and to choose the best one according to a cost function based on points cloud features. More recently, Boonvisut *et al.* [17] proposed an algorithm for the identification of the boundaries of deformable tissues, and to use it for both offline and online planning in robot-manipulation tasks. Moreover, Calli *et al.* [18] presented a dataset to test method for manipulation tasks.

Continuing in the same line, Jia *et al.* [19] presented a grasping strategy for non-rigid planar objects using just two fingers. This research assumes a linear elasticity model of the object surface. Afterwards Lin, *et al.* [20] used this same idea applied to grasp and lift 3D objects measuring when a secure grip is achieved under contact friction. Unlike both works, our proposal does not use a deformation linear model and the detection of deformations is realised using 3D visual features over the entire object surface not only the contact regions close to fingers. Moreover, our system can work with nonplanar objects as 3D objects using more than two fingers; specifically, we use a multi-finger hand [21,22]. In our work, the secure grip is achieved under a measurement control to reach an appropriated deformation level. In line with our work, Navarro-Alarcon *et al.* [23,24] proposed a vision-based deformation controller for robot manipulators. In those works, the presented method estimated the deformation of an objects using visual-servoing to measure the Jacobian matrix of features on the object surface. The authors used a sponge with small marks located on the surface. In contrast, our proposal is based on the estimation of deformations to control the grasping tasks without artificial markers and without a previous known deformation model [25]. We also test our visual perception method with many different household objects, not only a sponge, as it was done in [24]. Recent research that use visual perception for manipulation tasks with non-rigid are included in works carried out by Alt *et al.* [26] and Sun *et al.* [27], although both the underlying idea and its applicability are very different to our proposal. Thus, on the one hand, in [26] are simultaneously combined both haptic and visual sensors for navigation and manipulation tasks. The authors used a deformable foam road mounted on the gripper to measure visually a 1d stress function when there was contact with deformation. On the other hand, in [27] a visual perception pipeline based on an active stereo robot head was implemented for autonomous flattening garments. It made a topology analysis of each wrinkle on clothes surface in order to identify deformations.

## 3. Visual Surveillance System for Robot Grasping Tasks of Objects

**Figure 1:**
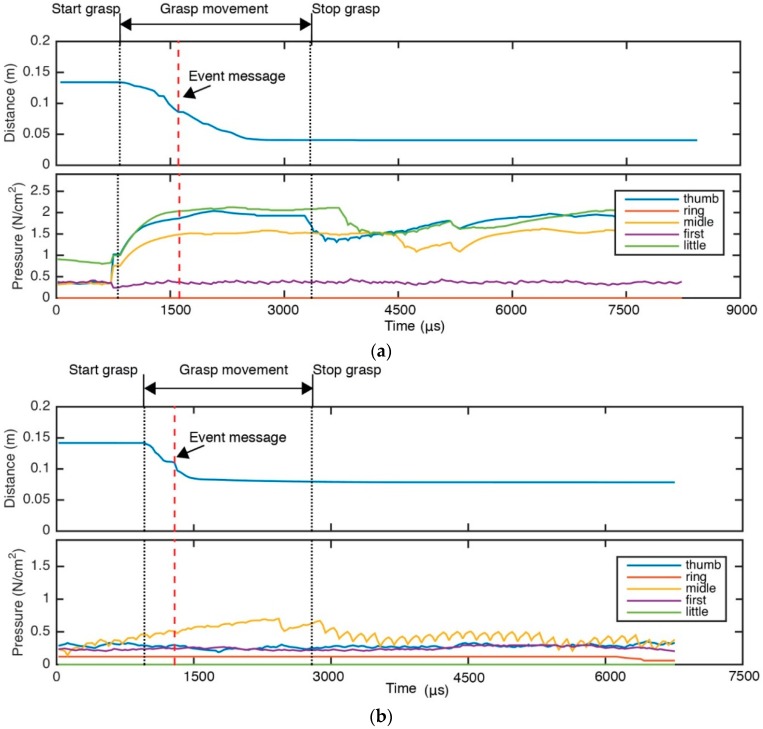
Comparison of different pressure for two real grasping tasks: (**a**) Pressure data allow to control the grasping; (**b**) Pressure is not valid to evaluate the grasping an object.

Modern approaches based on pressure data obtained from a tactile sensor may fail in complex grasping tasks (failure of the touch), in which the non-rigid objects change their shapes as their surfaces are deformed due to the forces applied by the fingers of a robot hand. In this case, the tactile sensors are often not able to obtain touch data correctly. Figure 1 shows the pressure of tactile sensors on grasping tasks of two different non-rigid objects. Figure 1a presents the pressure evolution for the grasping task of a brick. The pressure data retrieved from the tactile sensor are good enough due to that the pressures are greater than $0\text{ N}/{\mathrm{cm}}^{2}$ for four fingers and the values are greater than $1.5\text{ N}/{\mathrm{cm}}^{2}$ for three fingers. In contrast, Figure 1b presents the pressure evolution for the grasping task of a plastic glass. In this case the acquired pressure is not adequate. That is indicated by the fact the pressure values are close to zero for all fingers except the thumb ($0.5\;N/{\mathrm{cm}}^{2}$ is measured for thumb, but it is very low).

To overcome this problem, the novelty of our approach lies in that we use a visual sensing approach to obtain information about the object, such as shape, deformation and the interaction between the object and the robot hand. Therefore, our goal is to implement a visual system which serves as a surveillance module for the grasping control when there is no available tactile data, if, for example, the sensor is not working or fails due to its inability to measure pressure. In such scenarios, our visual system would be able validate if there is contact between the robot finger and the object.

**Figure 2:**
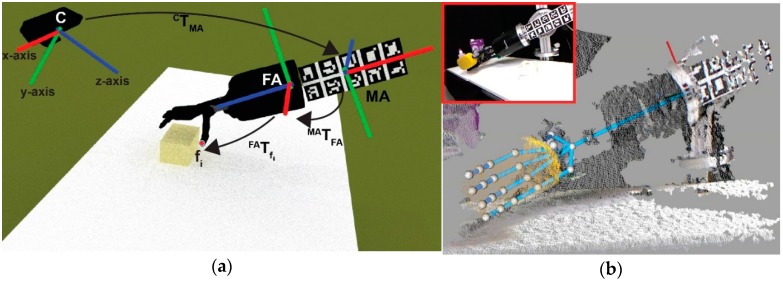
Workplace: (**a**) Scheme of the robot hand and RGBD sensor configuration; (**b**) RGB image and its point cloud associated with the robot hand and an object.

Considering the recent advances and availability of depth cameras such as RGBDs and the advances of the 3D processing tools for point clouds, we have designed a strategy that considers RGBD images as input data for the visual inspection of grasping tasks. To obtain visual data, a Microsoft Kinect sensor is used in this work. The Kinect sensor consists of a visual camera (RGB) and a depth sensor (an infrared projector and a camera). The Kinect sensor operates at 30 Hz and can offer images of 640 × 480 pixels but it has some weaknesses, such as problems with depth resolution and accuracy. The depth resolution decreases quadratically with respect to the distance between the sensor and the scene. Thus, if the camera is the world reference frame, the point spacing in the *z*-axis (Figure 2a) is as large as 7 cm at the maximum range of 5 m. In addition, the error of depth measurements (or accuracy) increases quadratically, reaching 4 cm at the maximum range of 5 m [28]. Moreover, another known problem is the unmatched edges, which is caused by pixels near to the object’s boundaries. In this case, wrong depth values are assigned by the Kinect sensor.

Our work assumes that the camera does not need to change its viewpoint because the robot hand is solely moved with according to the trajectory planning and grasp quality measure. Here, the sensor pose is static and is initially estimated according to the initial hand’s pose. It is not the objective of this research to find the best viewpoint of the camera for grasping tasks using the robot hand. The RGBD sensor is positioned according to previous works of other authors such as [29] in order to maximize the visible area of the manipulated object.

**Figure 3:**
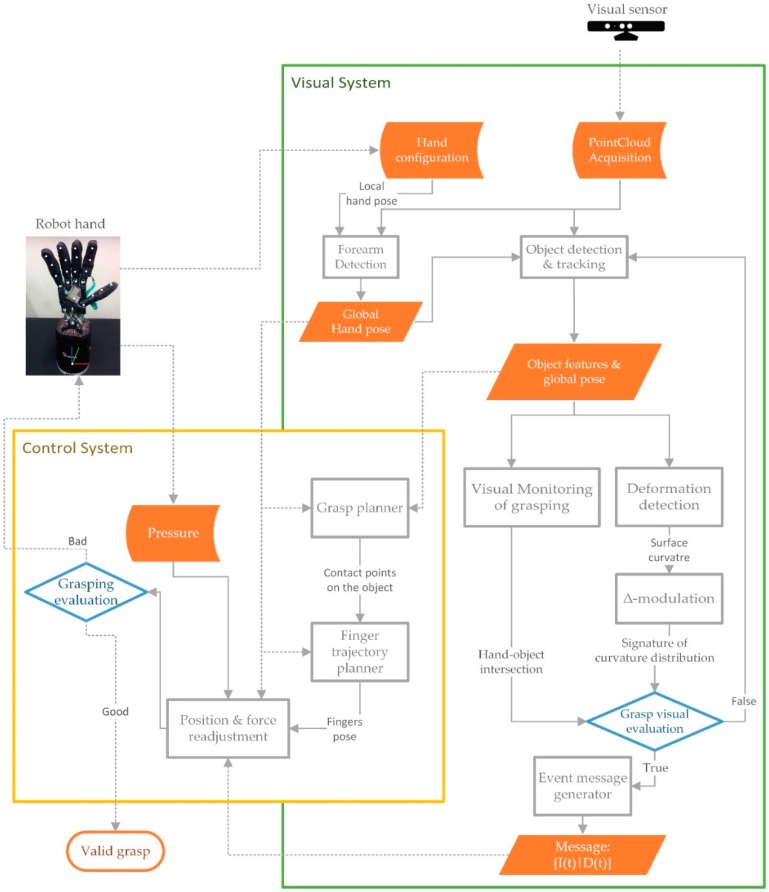
The overview of the system flowchart. The control system architecture (**yellow block**); and the proposed method for the visual system (**green block**).

The system was set up using ROS. The sensor is connected through a USB port to a computer which works as visual data server. Therefore, the visual system is ready to communicate with other systems such as the robot hand and tactile sensors. It should be noted that touch data from tactile sensors are not used as inputs or feedback of our visual system (Figure 3). It is the visual system which monitors the grasping tasks in which the tactile sensors can fail. Previously, Figure 1 has shown an example where the tactile sensor fails.

This work does not discuss how the control system works with the information received from the visual system, nor the readjustment of the fingers’ position nor the equilibrium during the grasping task, Although the control system used (yellow block in Figure 3) was presented by Delgado *et al.* in [30], this one implements a control system based on the kinematic model of the robot hand but without using the physical model of the manipulated object. Another approach of implementing a control system is presented in [8] (Chapter 2), which uses the physical model of the manipulated object.

On the one hand, the methods for determining contact points in a grasping process depend on whether the object is known or unknown. In the first case, fingertips contact positions are calculated as discussed in [31]. Here, Kragic *et al.* presented a real-time grasping planner to compute grasp points for known objects. To do this, is also required that the objects are detected and recognized by comparison of points cloud of object with models. We can use a surface descriptor-based method as is shown in our previous works [32] in order to describe the points cloud of object and to compare it with a surface model. In the second case, as is done in this work, the object is unknown then we extract the points cloud which represents the object located on a planar surface as a worktable by means of filtering of points cloud of scene and Random Sample Consensus (RANSAC). Thus, the points cloud of both object and table are separated. Afterwards, the centroid and the principal axis of points cloud of object are computed to approximate their position and orientation. The object normal vector is built using its centroid and the direction of the normal vector of the worktable. This way, the robot hand can be positioned and orientated for grasping. Later, the contact points for the thumb and middle fingertips are estimated by calculation of the orthogonal vector which intersects to the plane formed by the principal axis and the object normal vector.

On the other hand, the readjustment algorithm used in this work was presented in [33,34]. The algorithm receives as an input the finger joint trajectories and adapts them to the real contact pressure in order to guarantee that undesired slippage or contact-breaking is avoided throughout the manipulation task.

The contribution of this work is the visual system method and it is represented in the green block in Figure 3. This block presents a scheme of our method, based on data-driven 3D visual recognition. In particular, it shows the processing steps for the inspection of the grasping tasks where the robot hand starts moving forward until deformation is detected by the implemented method. The method is discussed in Section 4, Section 5 and Section 6. More specifically, our approach can be understood as a data-driven visual surveillance method that generates control events when potential grasping problems or anomalies are detected for the manipulated object—meaning significant deformations, slips or falls of the object that are caused by improper manipulation. Therefore, while the control system is working with the robot kinematics and tactile sensor, the visual system is working simultaneously, when it detects some of those anomalies in the grasping task, it sends an event message to the control system which determines a grasp quality measure and realizes the fingers movement planning (Figure 3). Figure 1 shows two examples were the visual system sends an event message before the control system finishes the grasping task.

The method is designed as a tracking loop with two pipelines (chains of processing blocks). The first one is composed by a processing block named “*visual monitoring of grasping*”. The second pipeline has two blocks named “*Deformation detection*” and “∆-*modulation*”. The method is completed when the conditional block named “*Grasping evaluation*” determines whether there has been a problem or whether the grasping task is realised. At the end of the method, the tracking loop sends an event message. This message is encapsulated as a data packet by the processing block named “*Event message generato”*. The event message contains data about object deformation level and object-hand intersection level. These processes are well explained in Section 4, Section 5 and Section 6. The remaining part of this section is focused on providing detail on the block named “*Object detection & tracking*”.

### 3.1. Object Detection

To carry on a tracking process, it is essential to carry out an initial object detection process. Let $I_{m}\left(r,g,b,d\right)$ denote the input data, the system has a point cloud P(x,y,z,r,g,b) by mapping the RGBD image from a calibration process of the visual sensor. The point cloud is partitioned into a set of disjointed regions. One of them represents the object to be manipulated, another is the robot hand and the rest is noise (data which are not considered). The green block of Figure 3 represents a complete overview of the proposed visual system in which the first time that process the “*object detection & tracking”* segments P into these regions. This process uses an object-recognition pipeline based on shape retrieval and interest region detections. There are several previous works that use these approaches, a case in point are [32,35], in which 3D objects are recognized and located on a worktable by using shape descriptors. Others, such as Aldoma *et al.* in [36], presented a review of current techniques of object recognition, comparing local and global mesh descriptors. Even though there are several works which use data sets of object mesh models for benchmarking in shape retrieval. These studies are not always helpful for manipulation experiments because they do not provide the physical properties of the objects, such as material stiffness or weight and how objects can change shape while they are being manipulated in real experiments. For this reason, our visual system (designed specifically for manipulation) is implemented considering that objects can be deformed over time.

The object recognition process is divided into the following stages:

1. Initialize the hand workspace
2. Get the object points
3. Select the object parts as super-voxels

**Figure 4:**
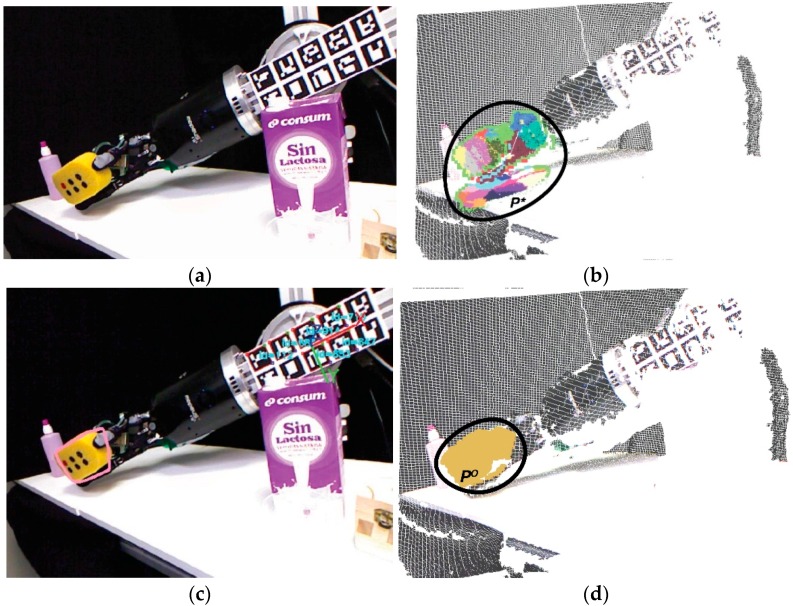
Samples of the object-detection process: (**a**) Original scene; (**b**) Colour and geometry segmentation process based on robot kinematics; (**c**) Clustering of regions to determine an object area; (**d**) Result of the 3D object detection.

The first stage initializes the hand workspace using the input data of two sets of sensors. They are internal sensors in the joints of the robot $S_{1}\left(E,t\right)$ and RGBD sensor $S_{2}\left(E,t\right)$. The goal of this stage is to obtain points in P(t) belonging to a points cloud that could potentially be part of the object $P^{*}\left(t\right)$ (Figure 4a,b). Both sensors are dependent on an environment E and time t as:

(1) $$S_{1}\left(E,t\right)=S_{1}\left(Q\left(t\right)\right)$$

where $Q=\left[f_{1}\;,..\;,\;\;f_{N}\right]$ is the pose for both the robot’s fingertips and palm, being $f_{i}=\left(q_{1}\;,..,\;\;q_{M}\right)$, which defines the joint parameters for each finger and the palm. In addition, $S_{2}\left(E,t\right)$ is defined as:

(2) $$S_{2}\left(E,t\right)=S_{2}\left(P\left(t\right)\right)$$

where  P(t) is a retrieved point cloud from the RGBD sensor at time t. From now on, P means P(t) for simplicity. Likewise, they work cooperatively, combining data to obtain the pose of a grasped object in the environment, which means that $S_{1}$ helps to $S_{2}$ to determine region $P^{*}$ from P. To do this, three sequential sub-processes are used: building the hand area; erasing noise; and sampling data. Firstly, a crop area placed in the geometric centre of the fingertips and palm kinematics is set; besides the radius of this area, this is two times the minimum distance between a fingertip and the palm position. Then, all points outside the crop area are removed. Secondly, the border, shadow and veil points are erased from the survivors’ points in the previous sub-process. In addition, the points around the position of the robot-hand links are erased. Thirdly, the resulting set of points is sampled using a voxelized strategy, with a voxel size of 2% of the minimum distance between a fingertip and the palm position.

The second stage obtains object points by means of a voxel cloud-connectivity segmentation method. This is used to determine better the object boundaries, as in Papon *et al.* [37]. Voxel Cloud Connectivity Segmentation (VCCS) is a variation of k-means clustering, with two important constraints: the seeding of super-voxel clusters is achieved by partitioning the 3D space and the iterative clustering algorithm enforces the strict spatial connectivity of occupied voxels. In short, VCCS efficiently generates and filters seeds according to how the neighbouring voxels are calculated. Each super-voxel cluster is represented with colours in Figure 4b. Finally, an iterative clustering algorithm enforces spatial connectivity. The statistical similarity test used here is Fisher’s test [38] in contrast with [37].

The third stage details the selection of super-voxels or clusters that belong to the manipulated object. This stage involves three sub-processes: selecting the seed cluster; iterating them via the neighbouring cluster; and merging the connected clusters. Firstly, the nearest cluster to the geometrical centre of the fingertips’ set and palm kinematics is selected as the seed cluster. Secondly, the remaining clusters are iterated to find newly connected clusters. Each new cluster is then labelled in line with how it should be merged. Thirdly, the labelled clusters are merged into the seed cluster, recovering the point cloud $P^{O}$ which represents the object from $P^{*}$ (Figure 4d).

Accuracy is increased in the segmentation process $P^{O}$ from $P^{*}$ due to the fact that $S_{1}$ helps $S_{2}$ to determine the region $P^{*}$ from P, instead of determining $P^{O}$ directly from P, as it would be done without the known kinematics of the robot hand. The reference frame for the robot kinematics is found using marker boards, as in [39]. This is done because the robot pose is unknown with respect to the world reference frame located in the RGBD sensor. Thus, once the forearm is located, we are able to obtain the pose of the robot’s fingertips and its palm as:

(3) TfiC=TMAC×TFAMA×TfiFA

where $f_{i}$ is the frame of a robot’s fingertips or palm,  C is the camera frame, MA is the marker board frame and FA is the forearm frame. If Tij denotes the transformation of the frame i with respect to the frame j, TMAC is the transformation of the MA w.r.t. the C, TFAMA is the the FA transformation with regards to the MA and the TfiFA is the transformation of a $f_{i}$ with respect to the FA. Thus, the transformation of each robot’s fingertips or palm frame w.r.t. the camera frame TfiC is given by the multiplication of these homogeneous transformations. Each homogeneous transformation represent rotations and translations.

### 3.2. Tracking Object Surface

The used tracking process is inspired by Fox’s works [40,41]. The author presents a statistical approach in order to increase the efficiency of particle filters by adapting the size of sample sets on-the-fly. The key idea of the author in these works is to bound the approximation error introduced by the sample-based representation of the particle filter. The measure used to approximate the error was Kullback-Leibler distance. This approach chooses a small number of samples if the density is focused on a small part of the state space. In another case, if the state uncertainty is high, it chooses a large number of samples.

Unfortunately, this approach presents undesirable characteristics to track points of object surface while this object is being manipulated; our work proposes to use the change in the number of sample as a tracking driver. This is that the tracked target (the set of points on object surface) is replaced when the number of samples changes, significantly. The idea is that the particle filter tracker is efficiency tracking when the number of particles is small. Thereby, the goal is to find when the number of these particles has dramatically grown up. Consequently, the method perceives that the object surface is being changed. Thus, the method detects this change on object surface by mean of the statistical relationship among the number of particles in the sample set over time as:

(4) $$\begin{array}{ccc} \Gamma \left(t\right)=\mu \left(t\right)+\sigma \left(t\right), & \mu \left(t\right)=\frac{\sum_{i=1}^{t}\gamma \left(i\right)}{t}, & \sigma \left(t\right)=\sqrt{\frac{\sum_{i=1}^{t}{\left(\gamma \left(i\right)-\mu \left(t\right)\right)}^{2}}{t}} \end{array}$$

where t is the time and γ(t) is the number of particles in the sample set at time t. μ(t) is the mean value and σ(t) is the standard deviation of the number of particles from t=0 until time t>0. Thus Γ(t) is the statistical relationship which determines when the target (the point cloud of surface object) must be replaced by the point cloud at time t−1. This approach sets up $P^{O}\left(t-1\right)$ as a new target when Γ(t−1)<γ(t). The efficiency of this strategy is dependent on how the method determines when the change on surface is significantly large to replace the tracked target with another point cloud more recent (at time t−1).

## 4. Visual Monitoring of Grasping

### 4.1. Modelling the Grasping Task like a Regional Intersection

**Figure 5:**
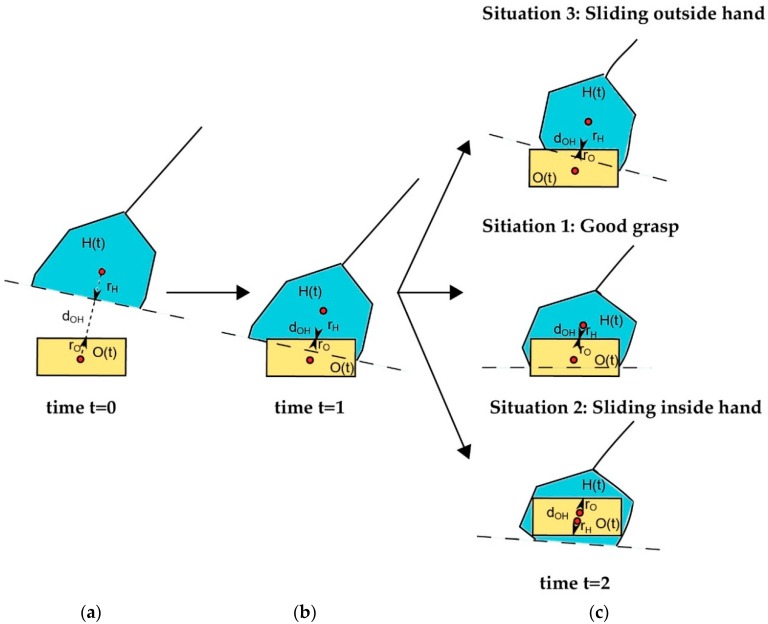
Scheme of the intersection between the robot hand and objects: (**a**) The hand volume has not yet intersected with the object volume; (**b**) The hand volume has started to intersect with the object volume; (**c**) Three final possible gasping situations.

One way to monitor and supervise the grasping tasks is to measure the interaction between items in the environment, the robot hand and a manipulated object. Our proposal is based on the visual relationship between the regions H(t) and O(t). Figure 5 illustrates three cases where this spatial information provides an interaction relationship between an object and the hand. At first, the object is not caught and the hand is not positioned on the object. At this stage, the fingers have not yet started to move and the tactile pressure should be zero (Figure 5a). When the hand is positioned according to the “*Grasp planner*” and “*Finger trajectory planne”* (Figure 3), the fingers are moved and some of them can press the object but it is not enough to grasp and lift the object without sliding (Figure 5b). Later, at a given time, the contact and appropriate touch of the fingertips allow the robot hand to pick up the object correctly, or not. That is, sometimes, if the object has flexible properties, then it can change its position due to the finger pressure, causing a sliding of the object among the fingers (Figure 5c).

### 4.2. Computing the Intersection between the Robot Hand and the Object

**Figure 6:**
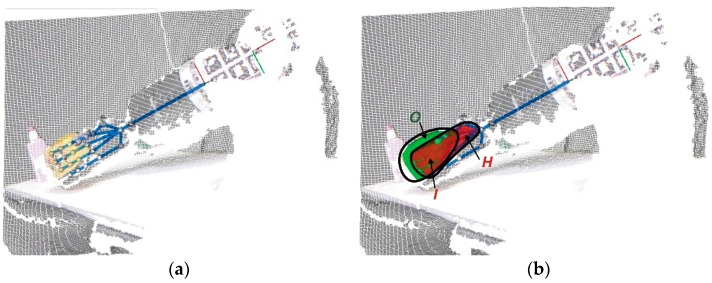
Visualization of the main steps of the visual monitoring algorithm for a grasped object scene: (**a**) point cloud of the scene and robot kinematics; (**b**) result of the detection of 3D regions of both the robot hand and the object.

The proposed method of “*visual monitoring of grasping*” supervises the grasping process and is based on an estimation of the intersection region. In any real grasping task (Figure 6a), the manipulated flexible objects O(t) change their volume and shape in relation to time t. Likewise, the robot hand H(t) changes its pose according to the kinematics. From now on to simplify, H means H(t) and O means O(t). Both the object and the hand are represented as 3D regions in this work. On the one hand, O is calculated using a segmentation process from the point cloud P of the scene acquired by the Kinect sensor (Figure 6), as discussed in Section 3. On the other hand, H is given by the hand pose and calculated from the kinematics of the robot (Figure 6a). We compute the 3D convex hull of the set of points of the object surface for O. Furthermore, we calculate a 3D virtual bounding hull that fits the points of fingertips and the palm area for the representation of H. The convex hull of a set of points is the smallest area which contains those points and its volume is calculated as:

(5) $$\left\{\sum_{i=1}^{\left|S\right|}\alpha_{i}p_{i}|\left(\forall i:\alpha_{i}\geq 0\right)\wedge \sum_{i=1}^{\left|S\right|}\alpha_{i}=1\right\}$$

where S is either H or O set and $p_{i}$ is a point of $P^{*}$. $\alpha_{i}$ is the weight assigned to $p_{i}$ in such a way that is a non-negative value if the point belongs to S. Thus, on the one hand, we have for each value $\alpha_{i}^{H}$ when S=H that:

(6) αiH(pi)={1|H|, pi∈Q×TfiC0, other case

where $p_{i}\in P^{*}$ is the point associated to $\alpha_{i}$, |H| is the number of points in H and Q×TfiC is the set of points indicating the fingertip poses. On the other hand, each value $\alpha_{i}^{O}$ when S=O is computed as:

(7) $$\alpha_{i}^{O}\left(p_{i}\right)=\{\begin{array}{c} \frac{1}{\left|O\right|},\;p_{i}\in P^{O}\\ 0,\;other\;case \end{array}$$

The interaction between the robot hand and the object is estimated by the relationship between the object and the robot hand, and it is computed by the comparison of the 3D regions H and O. In order to do this, we obtain the overlap between the 3D convex hull, object, and the virtual bounding hull, robot hand (Figure 6b). This is done as follows:

(8) $$I=H\cap^{\text{​}}O=\left\{\sum_{i=1}^{\left|I\right|}\alpha_{i}^{H}\alpha_{i}^{O}p_{i}|\left(\forall i:\alpha_{i}^{H}\alpha_{i}^{O}\geq 0\right)\wedge \sum_{i=1}^{\left|S\right|}\alpha_{i}^{H}\alpha_{i}^{O}=1\right\}$$

where I is the intersection volume between H and O. Therefore point $p_{i}\in I$ when at the same time $p_{i}\in H$ and $p_{i}\in O$. Moreover, the hand grasps the object when the intersection is greater than I(t)=O and smaller than I(t)=min(H(t), O(t)). Note that, I(t) is the generalization of I for the time t.

Finally, the grasping task is evaluated by the “*Grasp visual evaluation*” (Figure 3). This conditional block determines whether the visual system must finish and it generates an event message by “*Event message generator*” block or continues with the supervision of grasping task. Thus, when I(t)=0, and the object has fallen from the robot hand to the ground, “*Grasp visual evaluation”* finishes the tracking loop of the visual system and “*Event message generator*” block creates an event message encapsulated as a packet with the connect values of intersection and deformation. Hence this event message is sent to the control system.

## 5. Detection of Deformations by Means of Surface Curvatures

A novel contribution of this work is its presentation of an approach for the detection of deformation in flexible objects. The method “*detection of deformations*” is based on the general idea presented in [42], where the authors compute surface gradients for the modelling of surfaces as curvature levels, although the objective is much more ambitious in this work. Here, the issue goes further than a specific implementation of that general idea, insofar as we present a new method for detecting when a deformation is occurring in real time by means of differential deformation estimation among time points. This is done by analysing the curvatures of the object’s surface through a timing sequence.

The aim is to know whether the flexible object is grasped properly. That is to say, the object is considered it has been well grasped if the deformation distribution measured as a surface variation undergoes meaningful changes in connecting with a reference frame. The ideal situation is given when the distribution of surface variation is constant throughout the grasping task. The comparison process between the surface at various time points is conducted by comparing variations in the surface in each point of a points cloud which contains the object.

The method locally analyses the points cloud which represents the object surface for extracting the surface variation as curvature values $c_{p_{i}}$ at each point of the surface $P^{O}=\left\{p_{i}\in {\mathbb{R}}^{3}\right\}$. To analyse the surface variation at point $p_{i}$, the eigenvalues and the eigenvector of covariance $C_{P}$ in a neighbourhood environment with a radius r matrix are extracted as in [42]. $C_{P}$ is computed as:

(9) $$C_{P}=PP^{T}=\left[\begin{array}{c} p_{i_{1}}-\bar{p}\\ \cdots \\ p_{i_{k}}-\bar{p} \end{array}\right]{\left[\begin{array}{c} p_{i_{1}}-\bar{p}\\ \cdots \\ p_{i_{k}}-\bar{p} \end{array}\right]}^{T}$$

where each $p_{i_{j}}$ is a point of the neighbourhood environment $N_{j}$ and $\bar{p}$ is the centroid of the path. k defines the number of points $N_{j}$.

Besides, the computation of eigenvalues $\lambda_{j}$ and eigenvectors $v_{j}$ of $C_{P}$ is done by applying singular value decomposition (SVD) as:

(10) $$C_{P}\cdot v_{j}=\lambda_{j}\cdot v_{j}$$

Once the eigenvalues are computed, the surface variation (curvature) is calculated as:

(11) $$c_{p_{i}}=\frac{\lambda_{0}}{\lambda_{0}+\lambda_{1}+\lambda_{2}}$$

where $\lambda_{0}\leq \lambda_{1}\leq \lambda_{2}$ are the eigenvalues associated to the eigenvectors of a covariance matrix. Then, the points with similar curvature values $c_{p_{i}}$ are clustered together in level curves. These level curves are defined as a function $S_{P}:\;{\mathbb{R}}^{3}\to \mathbb{R}\;,\;$ as follows:

(12) $$S_{P}=\left\{\begin{array}{cc} \left(x,y,z\right)\in {\mathbb{R}}^{3}: & \Phi \left(x,y,z\right)=l \end{array}\right\}$$

where l is a constant value and represents a level curve on the surface. The control rule used to determine whether a specific point $p_{i}$ belongs to a specific level curve $l_{i}$ is:

(13) $$\left\{\;p_{i}\in l_{k}\;|\;k=curvature\left(p_{i}\right)\times \frac{\left|P^{*}\right|}{\left|P\right|}\;\right\}\;$$

Consequently, each of the level curves is computed as a cluster, and it is represented with the same colour when the points of surface have a similar value of the curvature (Figure 6a). Therefore, two significant curves are highlighted, such as the level of maximum curvature (displayed in dark blue) and the level of boundary curvature (in yellow), which represents all points with the minimum curvature, without considering the zero value (displayed in red is the region with a value of zero). If the curvature is zero, then there is no deformation and all points of the surface lie on a flat.

**Figure 7:**
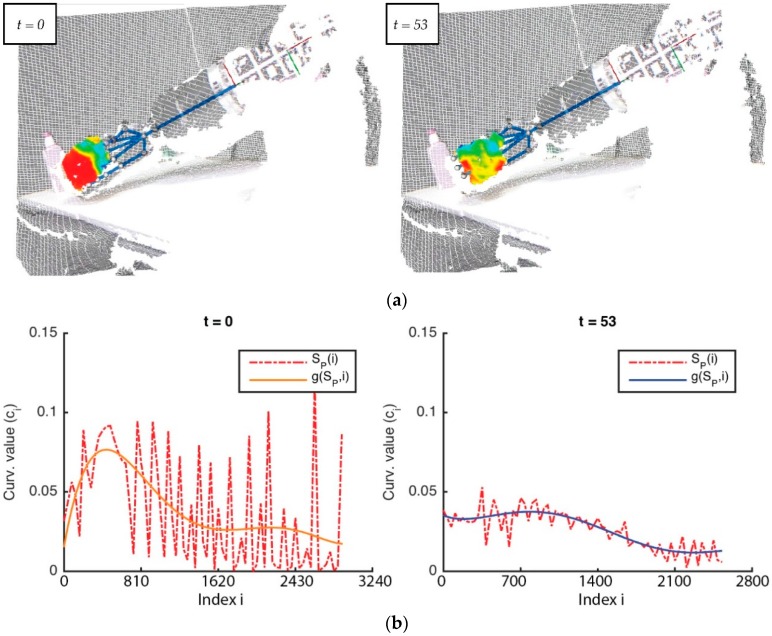
Two frames of the same manipulation sequence at two different time points: (**a**) The model of deformation computing the surface curvatures, grouped as level curves; (**b**) The signature of the curvature distribution.

Figure 7 represents $P^{O}$ and its level curves $S_{P}$ at two different time t; in this case, t=0 and t=53. It is later obtained via a signal which adjusts the original signature of the curvature distribution for $P^{O}$. This is done by using a least-squares fitting of the curvature values by means of a quadratic function g(f,x) that allows the method to obtain a more representative signature of the curvature distribution as:

(14) $$g\left(f,x\right)=w_{1}x^{n}+w_{2}x^{n-1}+\ldots +w_{n}x+w_{n+1}=\sum_{k=0}^{n}w_{k+1}x^{n-k}≅f\left(x\right)$$

where n is the range of the quadratic function and $w_{n}$ is a coefficient associated to the polynomial term x. Figure 7b represents $S_{P}$ and its adjust $g\left(S_{P},i\right)\;$ with a continuous curve line (the colour is orange in the first case, and blue in the second case) at two different time.

## 6. Δ-Modulation and Grasping Evaluation

The detection of deformation is a huge problem that cannot be tackled without regard to the time variable t, *i.e.*, the signature of the curvature distribution of an object, as is shown in Figure 7, gives information regarding its surface shape but not its deformation. Therefore, in order to obtain deformation data, the way as changes the surface shape with respect to the time was studied using our method of ‘Δ*-modulation*. Figure 7 is an example of how the signature of curvature distribution can be retrieved with regards to time, by comparing the curvature values between different time points. Figure 7, especially, shows the curvature and its Δ-modulation for the point clouds shown in Figure 6.

The method of Δ-modulation used to encode the signature of curvature distribution with regards to t in concrete is a sequence with two time values, for reducing its complexity. This method provides advantages for the comparison of signals over time. In Δ-modulation, the input signature is approximated by a step function where each sampling interval α increases or decreases one quantization level δ. Figure 7a,b both show an example in which the step function is overlapped with the original signature with regard to time. The main feature of the step function is that its behaviour is binary: at every α, the function moves up or down by the amount δ. Therefore, the Δ-modulation output can be represented by just one bit for each sample, and is represented in Figure 7a,b as a binary function. Consequently, Δ-modulation obtains a bit chain that approximates the derivative of the original signature. It is generated as 1 if the step function increases or 0 if the step function decreases.

**Figure 8:**
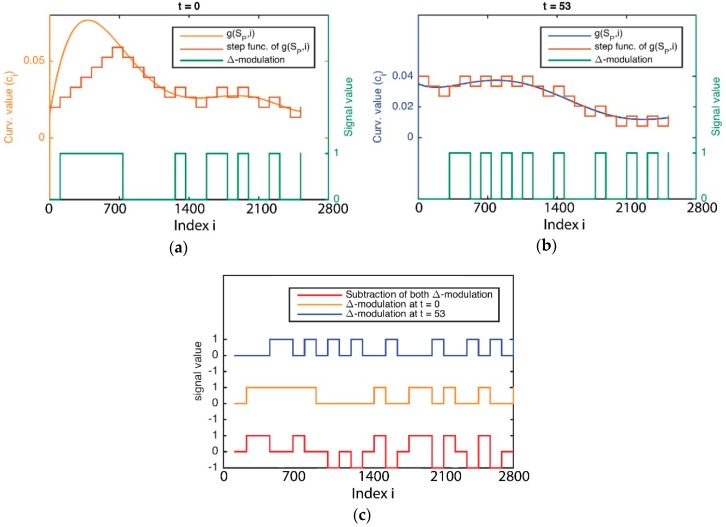
The delta modulation output and its approximation staircase signal for the curvature signature: (**a**) At time 0; (**b**) At time *t*; (**c**) The result of subtracting both modulated signals.

Once Δ-modulation has encoded the signature of a curvature’s distribution in both times, the outputs are compared by a subtraction logic operation as is shown in Figure 8c. A simple interpretation of this operation result is as follows:

- Case 1: The object is not deformed whether the output is always 0.
- Case 2: It is considered that the object is deformed.

**Figure 9:**
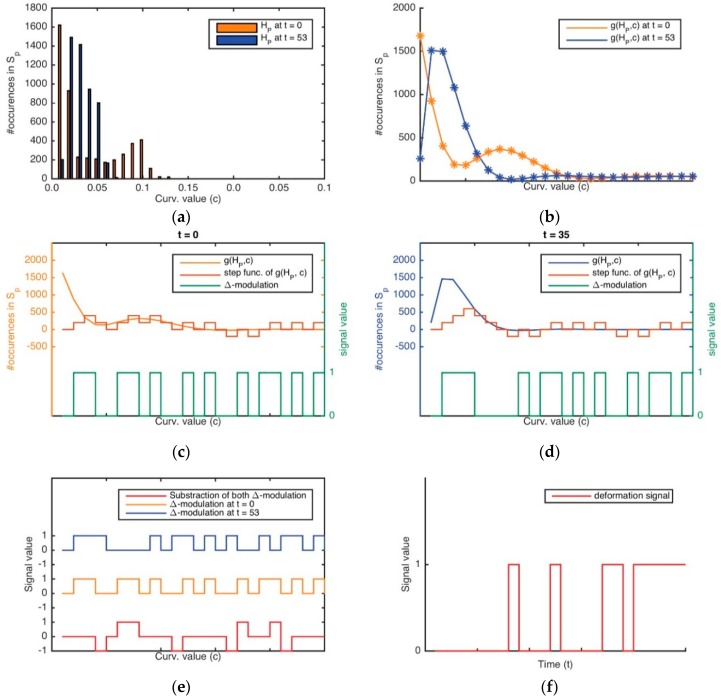
(**a**) The histograms of the curvature signatures of a point cloud, which represents an object, at two time points; (**b**) The signal of the histogram; (**c**,**d**) The delta modulation output and its approximation staircase signal for the signal of the histogram in both cases; (**e**) The result of subtracting both modulated signals; (**f**) The deformation signal measured as a time signal in a grasping task.

A disadvantage of this method is that it is dependent on the type of fitting used (here, a quadratic function), which could result in an over-fitting or under-fitting problem and then the function would not be sufficiently representative. To overcome this problem, we simplify the signature, making a histogram into a generating function that represents the signature of the curvature distribution in a simpler way that before (Figure 9a,b). The use of a histogram makes this method adjustable in terms of its sensitiveness. For instance, if we want a more reactive approach for deformation detection, it will be better the use of many classes. Therefore, we use this new representation of the signature, named curvature histogram $H_{P}$ in order to apply the “Δ*-modulation*” method and so we can easily acquire a derivative function which is comparable itself between two time points (Figure 9c,d). The binary comparison of both derivative functions is a new pulse function, as shown in Figure 9e,f. This signal is created by adding 0 in the context of “case 1”, or 1 in the context of “case 0”.

**Figure 10:**
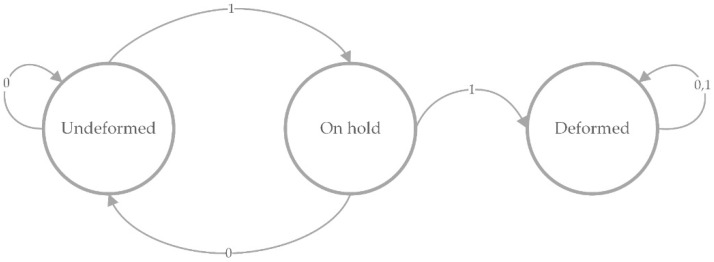
The Finite-State Machine for identifying from the deformation signal whether the object has been already deformed.

Furthermore, the proposed method is able to determine when the manipulated object is being deformed, implementing it as a Finite-State Machine (FSM). Figure 10 represents this implementation. The FSM starts in the “undeformed” state and remains in this state until it receives the value “1” from the deformation signal; then the FSM changes to the state called “on hold”. Once in the “on hold” state, the FSM returns to the previous state is named “undeformed”, or changes to another state called “deformed”, depending on whether the deformation signal represents a low or high level, respectively. This middle state is used to prevent the method from detecting false positives (a value of “1” that should be “0”) according to the deformation signal.

Aside from the evaluation of grasping by measuring of intersection I and deformation “*Grasp visual evaluation*” (Figure 3) is a conditional block to check which is the state of the FSM. Thus, “*Grasp visual evaluation*” finishes the tracking loop of the visual system and “*Event message generator*” block creates an event message, as it is commented in Section 4, if FSM is on “deformed” state.

## 7. Experiments and Results

In this section, we show the capabilities and effectiveness of our visual method. This method combines 3D visual processing from RGBD and the dexterous Shadow hand kinematics in order to accomplish the proper grasping of non-rigid objects. Our method implements the grasp adjustment from vision-based switching controller. In each step, the algorithm checks the interaction between the robot hand and the grasped object using visual information. The ability of our visual perception algorithm allows the robot system to correct the uncertainty/error caused by the lack of tactile/force data, or bad measurements when the grasped object has flexibility properties, such as in objects made from plastic polymers.

The next four subsections describe several relevant grasping experiments using flexible objects that were made from different materials, and that also have different shapes, sizes, textures and colours: a sponge, a brick, a plastic glass and a shoe insole. Each of those objects presents a challenge for the method. Some relevant frames of the manipulation task showing visual information computed by our system are presented for three of them, such as the result of the detected object tracker, the curvature data and the volume evolutions.

### 7.1. Experiment 1: Sponge

**Figure 11:**
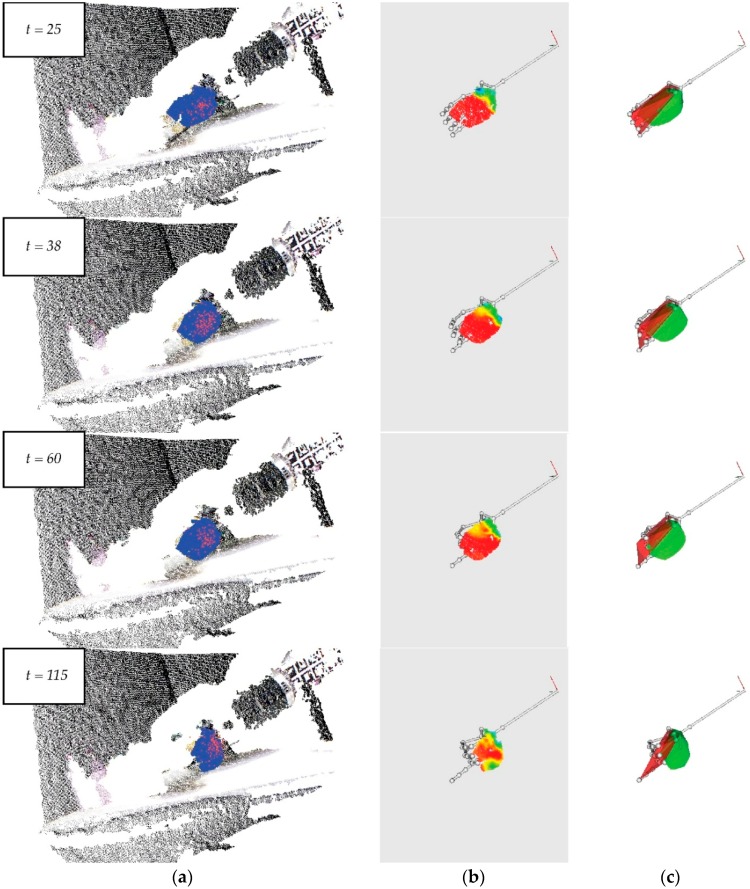
A sequence of video frames showing the grasping of a sponge: (**a**) The points cloud of the scene and the tracking process in the object’s detection; (**b**) A curvature map to measure the deformation level of the object; (**c**) The intersection for measuring the visual contact between the robot hand and object.

The sponge is ideal for testing the grasping tasks of flexible objects because it has a homogeneous distribution of elasticity and stiffness on its surface. These properties generate high levels of deformation close to attachment points where the fingers are located, but only slight deformations in the object’s centre (Figure 11a). For this reason, the sponge is rapidly deformed close to the fingertips at the beginning of the grasping process, although the deformation velocity tends to decrease quickly over time. Additionally, the observed deformation is regular and incremental regarding time (Figure 11b). The sponge’s homogeneous colour and regular geometry help the “object detection” module of our approach to extract the object’s region from the points cloud of the scene. It should be noted that the sponge’s size decreases gradually due to the properties mentioned above.

This test is a points cloud sequence of a grasping movement with 250 frames. The fingers of the robot hand do not move in the first 25 frames. The following frames show a sudden, sharp hand movement; this can be seen between Frames 26–115. Then, from frame 116 until the end, the hand movement is not considered relevant to this experiment because the deformation can be detected in the previous frames.

On the one hand, Figure 11a shows the tracking of the detected object. In particular, it shows how the target model of the sponge evolves dynamically during the deformation, fitting and tracking the object while its size is changing as a result of the contact force. On the other hand, Figure 11b shows the traces of the curvature map in which the regions with low curvature levels slowly disappear, but, simultaneously, the deformation regions close to the fingers indicate that the curvature levels are constant from frame 38 onwards. Additionally, Figure 11c shows the decrease in both the region of the hand and the object estimated as a hull.

### 7.2. Experiment 2: Brick

**Figure 12:**
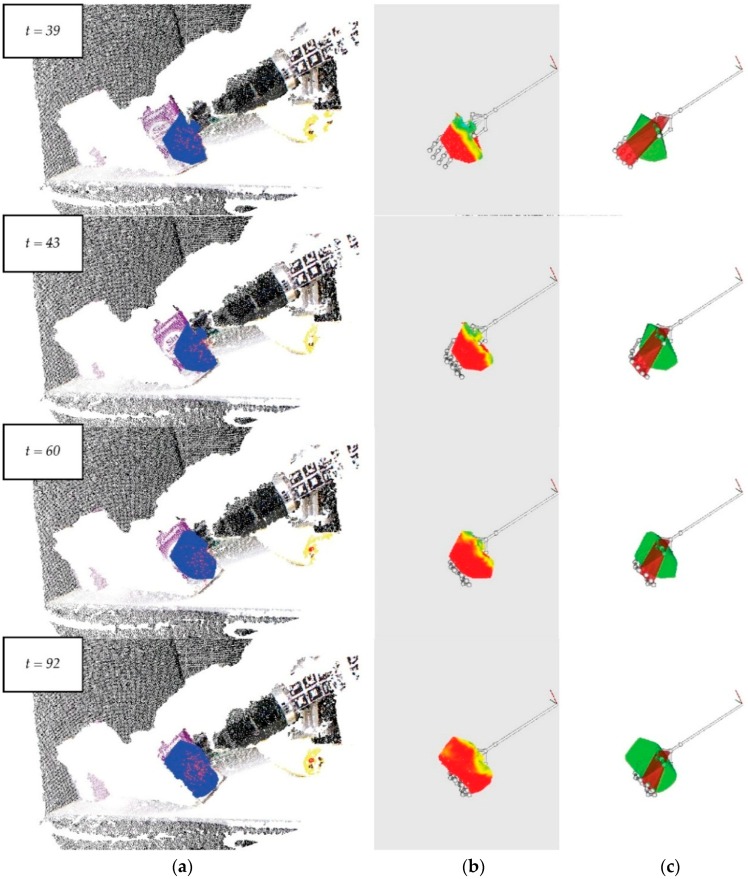
Some frames from a video sequence depicting a brick: (**a**) The points cloud of the scene and the tracking process in the object’s detection; (**b**) A curvature map to measure the deformation level of the brick’s surface; (**c**) The intersection to measure the visual contact between the robot hand and the object.

In contrast with the sponge’s features, the brick’s features are not homogeneous. The brick is made of several carton caps and their number, shape and structure determine the level of the brick’s elasticity, flexibility and stiffness. The most rigid points of its structure are its edges and the least rigid ones are the centre of the sides. These properties generate irregular deformation, limiting the spread of deformations to the brick’s edges (Figure 12b).

We have chosen a brick because it is a very common household object and has been used widely in recognition experiments by other researchers in the field. A brick is also usually painted with serigraphs of different colours. Its colour is not homogeneous and this fact entails more complexity and a new challenge for the object’s recognition and the tracking process (Figure 12a).

As above, the test includes 250 frames; however, the robot is motionless in the first 40 frames and it moves between Frames 41 and 60, in which it is possible to determine the brick’s deformation level and the interaction between the hand and the brick (Figure 12b,c). The other frames do not provide interesting information for the surveillance of this grasping task.

Figure 12a highlights the small amount of variability in the frames; this fact indicates the robustness of the object tracker. Besides this, Figure 12b shows slight variations of deformation in the first few frames but remains almost constant after the deformation. Additionally, the intersection changes only a little because of the brick’s rigidity (Figure 12c).

### 7.3. Experiment 3: Plastic Glasses

This experiment has been designed to show the behaviour of our approach when using another household object, such as a plastic glass. Its structure and manufacturing materials define new elastic and flexibility properties compared with those of the objects above. In particular, the grasping tasks cause irregular deformations of the glass. Its features of a homogeneous colour and simple geometry expedite the creation of a vision system for suitable recognition. As our aim is not to present new techniques for object recognition, we have always used objects that do not have complex shapes and many colours. In this case, the glass allows the vision system to check the behaviour of the newly implemented methods when the object size is smaller than a sponge or brick, and is made of another material.

**Figure 13:**
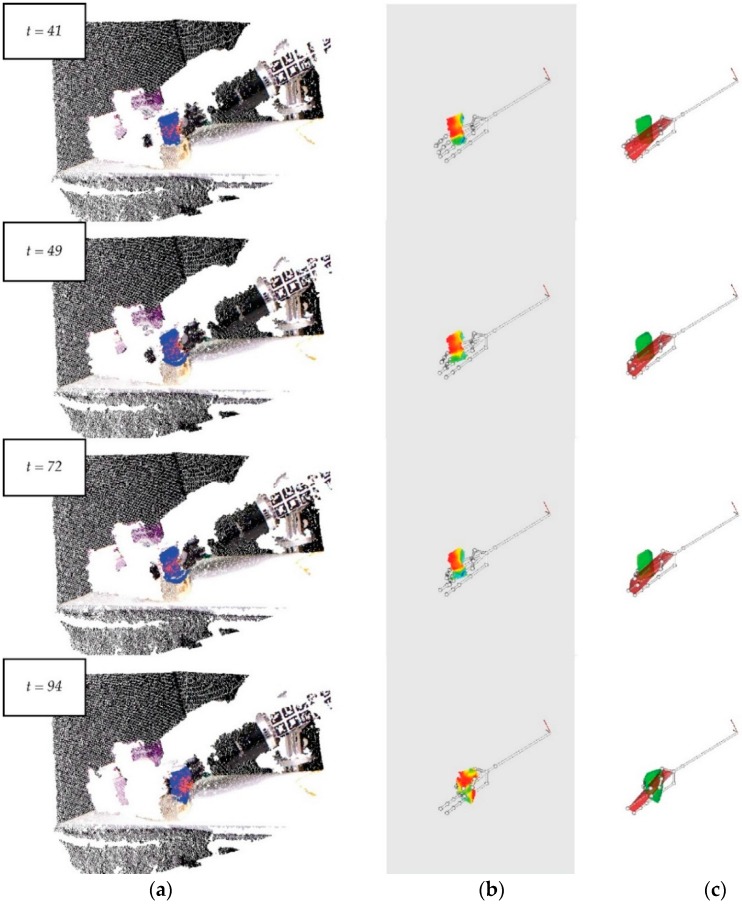
Some frames of a video sequence of a plastic glass: (**a**) The points cloud of the scene and the tracking process in the object’s detection; (**b**) A curvature map to measure the deformation level of the glass’s surface; (**c**) The intersection to measure the visual contact between the robot hand and the object.

The test also has 250 frames but, here, the first 18 frames show the robot hand when it is motionless, frames 19 to 38 show the robot making a little movement and from here until frame 133, the fingers make a grasping movement (Figure 13). As in previous experiments, the rest of the frames do not supply new data of interest for the detection of deformation and the control of grasping movements. In this experiment, little variability in the tracker’s evolution is observed in Figure 13a, in contrast with a great variability in the curvature map, shown in Figure 12b.

### 7.4. Experiment 4: Shoe Insole

**Figure 14:**
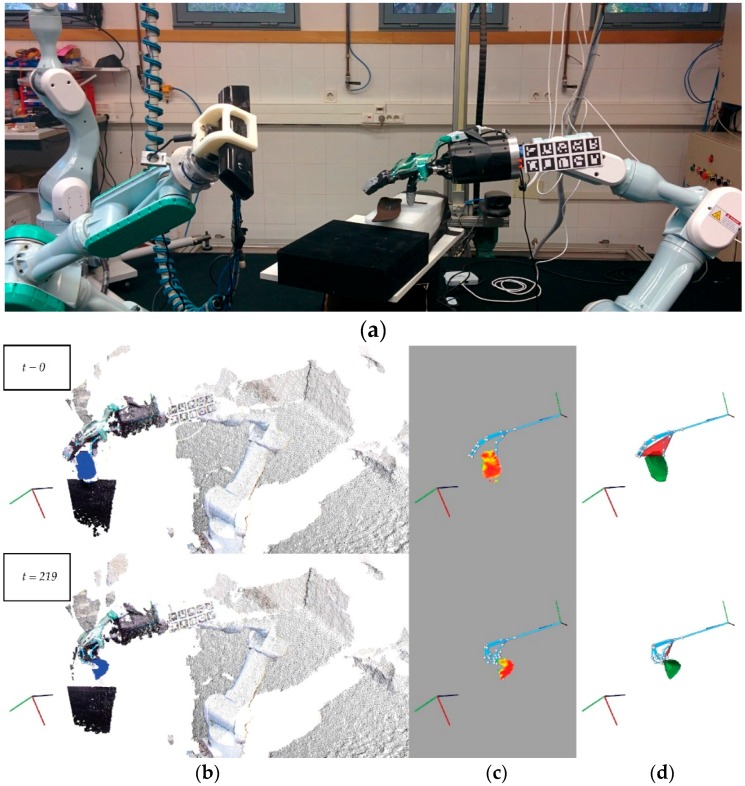
Some frames of a video sequence of a shoe insole: (**a**) An overview of the visual system working in a new workspace; (**b**) The points cloud of the scene and the tracking process in the object’s detection; (**c**) A curvature map to measure the deformation level of the glass’s surface; (**d**) The intersection to measure the visual contact between the robot hand and the object.

The last experiment was carried out in a different environment with a different ambient lighting. In contrast to the three previous experiments, here, both robot hand and RGBD sensor are mounted at the end of industrial robot arms (Figure 14a).

Then, the recognition process of search a marker board is no longer necessary since The fingers pose are calculated from the spatial location of the industrial robot arm which is equipped with de RGBD sensor as:

(15) TfiC=TRC×TER×TFAE×TfiFA

where R is the robot frame located at its base and E is the effector-robot. TRC×TER are the transformation of the robot frame with respect to the camera and the transformation of the effector-robot with respect to the robot frame, respectively.

This experiment (Figure 14b) has 250 frames as above experiments. By comparison with previous Experiments 6.1–6.3, the main challenge of this experiment is to prove that our visual system works when a scenario is different. Here, hand-robot is mounted at the end of industrial robot, the ambient lighting has more intensity, the pose of camera has also changed and the object is another.

## 8. Discussion and Analysis of the Results

**Figure 15:**
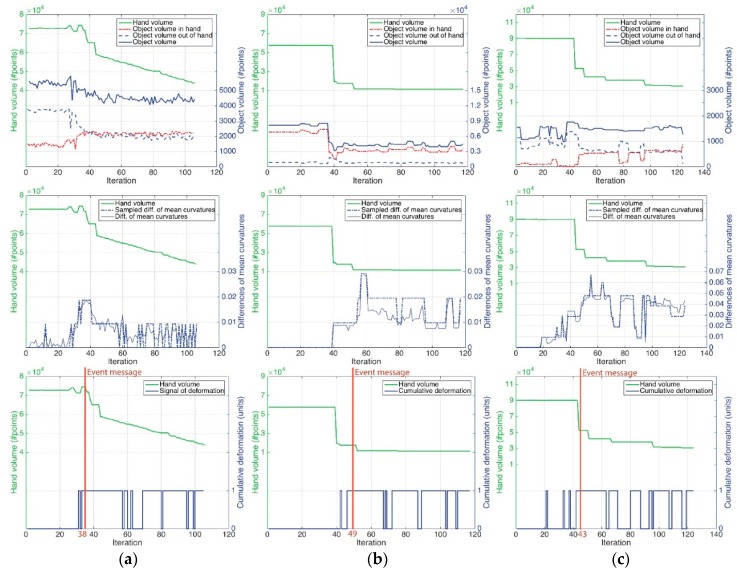
Grasping and deformation results: (**a**) Sponge; (**b**) Brick; (**c**) Plastic glass.

The previous experiments, shown in Figure 11, Figure 12 and Figure 13, were analysed to determine the main capability of how well our visual monitoring strategy works in discovering deformations and determining a good grasping motion. This goal was achieved by the two new methods presented here. First, the proposed method of “*detection deformation*” with “∆*-modulation*” is able to measure deformations with regards to time, from RGBD data using a curvature distribution. The second method of “*visual monitoring of grasping*”, based on the intersection of regions, is used to determine whether an object is being correctly grasped, with the absence of contact between the robot hand and the object. The results of this analysis are shown in Figure 15.

For each experiment, as it is noted above, the results of the visual monitoring approach are shown in the first row of the figure. The charts show the relationship between the H(t) and O(t) volumes, as well as I(t) and O(t)−I(t). It is clear that H(t) tends to decrease due to the hand’s movement. This is a typical case of a grasping task in which the robot hand is closed in order to wrap around and grip an object. In contrast, O(t) does not follow that same trend. For example, in the case of the sponge, its volume does not change much. This fact is due because the area of the object located close to the fingers is compressed and so the other parts located further away are expanding. Furthermore, these charts show the grasp-monitoring results. Thereby, if the red dashed lines I(t) tend towards zero, then the hand is losing its grip on the object. Consequently, O(t)−I(t) tending towards O(t) means the same thing. Apart from that result, the second row of the figure shows how the average of the curvature distribution changes over time. These curves show the relationship between the average of the curvature distribution at the initial time and the rest of time t, ensuring the consistency of the results shown in the third row of Figure 15.

Moreover, the results of our method “*deformation detection*” with “∆*-modulation*” are shown in the third row of Figure 15. The three plots present a view of how the deformation signature evolves in connection with H(t), *i.e.*, how the object is deformed while the hand is working. These results are used to analyse the sensitivity, specificity and accuracy (Table 1) of our approach for detecting deformations by using the hand’s movement. Three sequences for each object (nine tests) with 250 frames each one was used to carry out this study.

The three statistical features give information about the behaviours of our method. The sensitivity measures the proportion of times that the visual system has determined correctly that the object is deformed. The sensitivity is computed as:

(16) $$sensitivity=\frac{TP}{P}=\frac{TP}{TP+FN}$$

where P is the number of times that the system detects a deformation, in contrast, N is the number of times that the system does not detect deformation.Then TP is the number of times that the system succeeds about deformation and FP is the number of times that the system fails detecting deformations. Similarly, TN is the number of times that the visual system succeeds when this determines if the object is not deformed and FN is the times that the visual system confuses an undeformed object with a deformed object. Using this relation, the specificity is defined as:

(17) $$specificity=\frac{TN}{N}=\frac{TN}{FP+TN}$$

In contrast with the sensitivity, specificity measures the proportion of times that the visual system has determined correctly that the object is not deformed. Also, the accuracy is calculated in this work as:

(18) $$accuracy=\frac{TP+TN}{P+N}$$

Ideally, the behaviour of the deformation signal in these experiments should be like a step function with just one rising edge, since the robot hand makes only one grasping movement. This rising edge would have to occur in the moment at which the hand starts to grasp the object and its H(t) starts to change. Hence “1” values before the ideal rising edge are considered to be false positive “0” values; after that, they are considered to be false negative ones.

## 9. Conclusions

The use of visual data in grasping tasks and intelligent robot manipulation is still an emerging topic. In the past, force and tactile sensors have often been used for these tasks without considering visual information. Although there are some approaches that use some visual data, they were just designed to recognize the object to be grasped or manipulated but never to check or supervise the task during the grasping process in order to detect when deformations occurred, or when there was a loss of contact if it was caused by displacement of the object within the hand. In this work, the experiments focused on 3D sensors, such as RGBD, combined with a multi-fingered robot hand, without considering tactile data from another kind of sensor. Furthermore, there are still some challenges remaining when the grasping tasks are directed to solid objects as much as to elastic ones.

The proposed approach is motivated by the need to develop new strategies to solve problems throughout the grasping tasks and to supply robustness. Thus, this paper presents a novel sensorized approach in order to carry out robot-hand manipulation of an unmarked object whose flexibility properties are unknown. This new algorithm is based on geometric information of the object and the curvature variations on its surface, and it is used to procure suitable grasping motions even when the object is being deformed. Using visual data with the help of robot-hand kinematics, this new approach allows us to check when a deformation is being caused by the multi-fingered robot and whether there is a lack of contact between the hand and object from a visual sensing point of view. The experiments show the behaviour of the methods in several grasping tasks in which the object’s deformation can be measured using a visual sensor. In the future, our approach could be combined with a hybrid tactile/visual control system for a reactive adjustment of pressure and the contact of fingers, whenever tactile data are not adequate for the manipulation process.

## Figures and Tables

**Table 1:** Information retrieved from the charts of Figure 15 for 3 tests of each object were carried out with different hand poses.

Experiment	Sensitivity ^1^ Average	Specificity ^2^ Average	Accuracy ^3^ Average
Tests 1–3: Sponge	0.7750	1	0.8286
Tests 4–6: Brick	0.8333	1	0.8889
Tests 7–9: Plastic glass	0.8837	0.7160	0.7742

^1^ Sensitivity measures the proportion of positives that are correctly identified as such; ^2^ Specificity measures the proportion of negatives that are correctly identified as such; ^3^ Accuracy is the level of measurement that yields true and consistent results.
